# Nonselective beta-adrenoceptor blocker use and risk of Parkinson’s disease: from multiple real-world evidence

**DOI:** 10.1186/s12916-023-03122-z

**Published:** 2023-11-14

**Authors:** Zeying Feng, Qiuping Zhao, Jingjing Wu, Yiping Yang, Xinru Jia, Junlong Ma, Haibo Tang, Hong Yuan, Guoping Yang, Yao Lu

**Affiliations:** 1 Clinical Trial Institution Office, Liuzhou Hospital of Guangzhou Women and Children’s Medical Center, Liuzhou, China; 2 Clinical Research Center, The Third Xiangya Hospital, Central South University, 138 TongZiPo Road, Changsha, 410013 Hunan China; 3 Fuwai Central China Cardiovascular Hospital, Heart Center of Henan Provincial People’s Hospital, Zhengzhou, China; 4 Department of Metabolic and Bariatric Surgery, The Third Xiangya Hospital, Central South University, Changsha, China; 5 XiangYa School of Pharmaceutical Sciences, Central South University, Changsha, China; 6 Schools of Life Course Sciences, King’s College London, London, UK

**Keywords:** Antihypertensive agents, Parkinson’s disease, UK Biobank, Real-world study

## Abstract

**Background:**

People with hypertension have a higher risk of developing Parkinson’s disease (PD), epidemiological evidence suggests that multiple antihypertensives may affect the occurrence and development of PD with inconsistent results. With multisource data, we sought to determine whether specific antihypertensive classes elevated or reduced the risk for PD.

**Methods:**

We used a mixed methods approach that combines 4 methodologies. First, we conducted a disproportionality analysis using the reports causing adverse events in the US Food and Drug Administration Adverse Events Reporting System (FAERS) to explore the effect of different classes of antihypertensive medications on the risk of PD; based on the findings from FAERS, a meta-analysis and a UK Biobank cohort analysis were used to further assess the association of drug use with PD; finally, we employed Mendelian randomization (MR) analysis to validate the causal relationship between the drug target and the occurrence of PD.

**Results:**

In the disproportionality analysis using the FAERS (*N* = 187,266), nonselective beta-adrenoceptor blockers (NBBs) were demonstrated to have a significant association with PD (reporting odds ratio (ROR) = 3.13; 95% CI 2.33–4.22). In the meta-analysis of 12 studies with 12,183,809 participants, PD risk was elevated in NBBs (RR, 1.64; 95% CI, 1.19–2.09) when stratified by subtypes of BBs. Among the 105,763 participants included in the cohort analysis using data from the UK Biobank, individuals who used NBBs had a significantly increased risk of PD compared to nonusers (HR, 1.47; 95% CI 1.04–2.06). The MR analysis revealed a significant association between higher expression of the β2 adrenergic receptor (ADRB2) gene, a drug target blocked by NBBs, and a reduced risk of PD (OR, 0.85; 95% CI 0.73–0.99).

**Conclusions:**

Our comprehensive study indicated that regular NBB use is associated with an increased risk of PD. In light of the detrimental effects of NBBs on PD, some people should choose alternative antihypertensive treatments.

**Supplementary Information:**

The online version contains supplementary material available at 10.1186/s12916-023-03122-z.

## Background

Parkinson’s disease (PD) is one of the most common neurodegenerative disorders, with a prevalence of PD exceeding 1% in the population 65 years of age and older and up to 5% in those 85 years of age and older, which creates a huge burden on public health. Currently, there is no cure for PD, and the available therapeutic options only result in a partial improvement of symptoms [1, 2]. At least 50% of nigrostriatal neurons have already been lost by the time the clinical diagnosis of PD is made [3]. Patients with hypertension are reported to have a 60–90% higher risk of developing PD [4–6]. Additionally, hypertension is one of the most common chronic diseases globally, especially in the elderly population. Consequently, the early identification and intervention into the factors that influence PD in patients with hypertension is of the utmost importance.

In recent years, the involvement of antihypertensive therapy in the pathogenesis of PD has attracted attention. Cell and animal studies suggest that angiotensin-converting enzyme inhibitors (ACEIs), angiotensin receptor blockers (ARBs), and calcium channel blockers (CCBs) might have neuroprotective effects on PD [7, 8]. On the other hand, a human cell model showed that treatment with beta-adrenoceptor blockers (BBs) led to a significant increase in the expression of α-synuclein (SNCA) mRNA levels and elevated α­synuclein protein concentrations, which might facilitate the development of PD [9]. Epidemiological studies have examined the associations between antihypertensive agents and the risk of PD; however, the results have been inconsistent [10]. Considering the widespread use of antihypertensive drugs and their potential as an intervenable target, it is of great interest to determine their impact on the development of PD in hypertensive patients.

Here, we intend to provide a comprehensive evaluation of the effect of antihypertensive drugs on PD by combining multiple real-world data. Pharmacovigilance data can be used to discover ADR signals. Meta-analysis is a crucial information source for evidence-based medicine and clinical decision-making since it summarizes evidence; UK Biobank provided an additional information source to enhance the credibility of the conclusions; Genome-wide association studies (GWAS); and Mendelian randomization analyses offer an opportunity to further clarify the causal relationship and underlying mechanisms linking antihypertensive drugs and PD.

## Methods

We used a mixed methods approach when combining 4 methodologies that included a disproportionality analysis employing the US Food and Drug Administration Adverse Event Reporting System (FAERS), a meta-analysis based on available observational studies, an observational study with UK Biobank data, and a drug target-based Mendelian randomization (MR) analysis. First, we conducted a disproportionality analysis to examine the association of different classes of antihypertensive medications on the risk of PD; based on the findings, a meta-analysis and a UK Biobank cohort analysis were used to further assess the association; finally, we used MR analysis to verify the causal relationship between the corresponding drug targets and PD (Additional file 1: Fig. S1).

### Pharmacovigilance data analysis

#### Data collection and definition

An open tool for cleaning and normalizing pharmacovigilance data from the FAERS website, Open Vigil 2, was used to query the data in this investigation [11]. We collected reports between the first quarter of 2004 and the first quarter of 2022 suspected of causing adverse events. For the present study, we only included patients with hypertension to mitigate confounding by indication. Cases were all reports of PD identified using the Medical Dictionary for Regulatory Activities (MedDRA) version 25.0 with the term “Parkinson-like events” as classified in the Standardized MedDRA Queries. “Noncases” were all other reports during the same period. Duplicate reports were excluded. Drug exposure was antihypertensive agents defined as “suspected” or “concomitant” in the reports. Antihypertensive agents were classified according to the Anatomical Therapeutic Chemical (ATC) classification system. The ATC categories of antihypertensive drug treatment considered were angiotensin-converting enzyme inhibitors (ACEIs) (code C09A), angiotensin receptor blockers (ARBs) (code C09C), BBs (code C07A), calcium channel blockers (CCBs) (code C08A), and thiazide diuretic agents (code C03A). BBs are classified into the nonselective beta-adrenoceptor blockers (NBBs) (code C07AA) and selective β1-adrenoceptor blockers (SBBs) (code C07AB) subclasses.

#### Statistical analysis

The risk of PD was calculated using the reporting odds ratio (ROR), which is the exposure odds ratio among reported cases of PD compared to the exposure odds ratio among reported noncases. A ROR > 2 indicated a significant association with the risk of developing PD according to the criteria of Evans [12].

### Meta-analysis

#### Literature search and study selection

Two authors (JJW and ZYF) independently searched PubMed, EMBASE, and Web of Science for pertinent studies published in English from the establishment of the database to December 31, 2022. The search strategies were a combination of keywords related to BBs and PD. Additional file 1: Table S1 provides the detailed search strategies. We conducted the meta-analysis under the Preferred Reporting Items for Systematic Reviews and Meta-Analysis (PRISMA) guidelines and prospectively recorded the data in the PROSPERO database (CRD42022351224). The Risk Of Bias In Non-randomized Studies of Interventions (ROBINS-I) was used to assess and score the methodological quality of the selected studies [13]. Two authors (JJW and ZYF) independently classified the certainty of evidence according to the approach of the GRADE working group [14]. All researchers discussed and settled any differences in the assessment results.

#### Data analysis

Given the expected heterogeneity between studies due to different study designs, interventions, exposure periods, subclasses of antihypertensive agents, and study participant characteristics, we performed a random-effects analysis with the Restricted Maximum Likelihood (REML) estimator to generate pooled effect estimates from the eligible studies, which allowed us to account for both between-study as well as within-study variation. We calculated the pooled risk ratio (RR) and corresponding 95% confidence interval (CI) to describe the association between agents and PD. Heterogeneity across studies was assessed using the *I*^2^ statistic, with *I*^2^ > 50% indicating that the heterogeneity was statistically significant. Additionally, we performed a leave-one-out analysis in which studies were systematically excluded one at a time to assess the influence of individual studies on the overall estimate. The forest plot was used for the graphical display of the results from the meta-analyses.

### A prospective UK biobank cohort study

#### Study participants

The UK Biobank cohort recruited over 500,000 middle-aged participants at 22 assessment centers in the UK between 2006 and 2010 [15]. When recruited, participants consented to provide blood, urine, and saliva samples at their nearest assessment center, as well as detailed information about sociodemographic, lifestyle, environment, medical history, and health-related factors via touchscreen and face-to-face interviews. For the present analysis, we excluded patients who did not use any antihypertensive agents at baseline. Additionally, participants with a pre-existing diagnosis of PD based on the International Classification of Diseases (ICD-10) code (G20) before the baseline assessment were excluded. The participant flow chart is described in Additional file 1: Fig. S2.

#### Exposure and outcome

Participants were assessed via touchscreen questionnaires and then verbally interviewed by trained staff to confirm the regular use of BBs. Regular users were defined as those who had been using the drug most days of the week for the last 4 weeks. BBs were defined according to the ATC classification system code C07A, and NBBs were classified using the ATC classification system code C07AA. According to the ICD 10 code (G20), we identified diagnosed PD cases in the UK Biobank inpatient data. The date of diagnosis was set as the first recorded date of PD codes recorded.

#### Assessment of covariates

Age, sex, ethnicity, smoking status, alcohol consumption, and educational level were self-reported at baseline. The Townsend deprivation score was provided directly in the UK Biobank dataset. The body mass index (BMI) was calculated by measuring height and weight by trained research staff. Considering the unclear relationship between BMI and the occurrence of PD [16, 17], we used Restricted Cubic Spline (RCS) curves to identify that BMI level with the HR = 1 as cutoff value. In subsequent analyses, we included BMI as a categorical variable in the model (BMI below 25.11; BMI between 25.11 and 28.63; BMI above 28.63). The patient’s comorbidities were identified based on the ICD-10 using the UK Biobank inpatient data [15]. Considering that long-term chronic diseases are associated with PD, we included the number of long-term conditions as covariates. The detailed definitions of long-term conditions are shown in Additional file 1: Table S2. We assessed the genetic susceptibility to PD by utilizing the polygenic risk score (PRS), which measures the combined influence of common genetic variants on the disease’s onset. The detailed method about PRS is presented in Additional file 1: Text S1, Fig. S3 and Fig. S4.

#### Statistical analysis

The Cox proportional hazards model was used to estimate the association between developing PD and all BBs. Subsequently, we focused our study on the relationship between NBBs and PD. Model 1 was adjusted by age at recruitment, sex, and ethnicity. Additionally, Model 2 was adjusted by PD risk factors that were prioritized, including smoking status, alcohol use, BMI, anxiety, socioeconomic status (Townsend deprivation score), income level, and educational level. Model 3 was further adjusted for hypertension, the duration of hypertension, diabetes, hyperlipidemia, cerebrovascular diseases, and the PRS for PD.

To check the robustness of the primary results, we performed several sensitivity analyses. First, to minimize reverse causality, we excluded participants who developed PD during the first year of follow-up. Second, considering that propranolol, a kind of NBB, is used for essential tremors that are associated with PD, our analyses deleted patients with tremors before their PD diagnosis. Third, we conducted the analysis adjusting for the number of long-term conditions. In addition, a competing risk regression was performed, considering all-cause mortality as a competing event.

### Mendelian randomization analysis

#### Data sources

Candidate genetic variants of outcome (PD) were obtained from the International Parkinson’s Disease Genomics Consortium (33,674 cases and 449,056 controls) [18]. As for exposures, we searched the eQTLs summary-level data available from eQTLGen Consortium (https://www.eqtlgen.org/). *P* value < 5 × 10^−7^ and r^2^ < 0.1 were used to filter available instrumental variables. The exposure and outcome data were loaded and harmonized using the defaulting setting.

#### Statistical analysis

To identify the causal effects of β1 adrenergic receptor (ADRB1) or β2 adrenergic receptor (ADRB2) expression with PD, we conducted a two-sample MR analysis using the “TwoSampleMR” R package (version 0.5.6). Since different MR methods have different sensitivities to different potential confounders, accommodate different scenarios, and vary in their statistical efficiency, we considered a range of MR methods. The primary analysis was conducted with random-effects inverse-variance weighted (IVW) method. We repeated analyses using alternative methods—weighted median and MR Egger estimators—which provide different degrees of robustness to bias from genetic pleiotropy.

## Results

### Disproportionality analysis using the FAERS

**Fig. 1:**
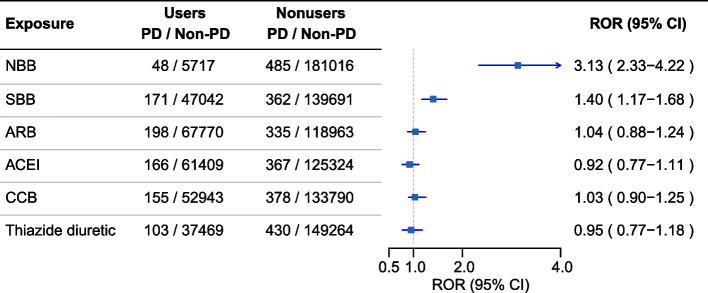
Reporting odds ratios (ROR) for the association between reports of PD and use of different classes of antihypertensive drugs. ACEI, angiotensin-converting enzyme inhibitor; ARB, angiotensin receptor blocker; CCB, calcium channel blocker; NBB, nonselective beta-adrenoceptor blockers; SBB, selective beta-1 receptor antagonist; PD, Parkinson's Disease; ROR, reporting odds ratio; CI, confidence interval

Among 187,266 reports from hypertensive patients submitted during the study period, the number of cases related to PD was 533. The disproportionality analysis, conducted at the level of the Standardized MedDRA Queries (SMQs), found a ROR of 3.13 (95% CI 2.33–4.22) for NBB use with “Parkinson-like events,” which suggested a statistically significant association with PD (Fig. 1) [12]. There was no signal which would indicate an association between the use of other antihypertensive agents and the risk of PD.

### Meta-analysis

In total, 2962 potentially eligible articles were identified: 280 from PubMed, 1754 from Embase, 928 from Web of Science, and 2664 were obtained after removing duplicates. Finally, of the 33 full-text articles assessed, 12 studies with 12,183,809 participants were considered for meta-analysis (Additional file 1:Fig. S5) [9, 19–29]. The characteristics of the included studies are summarized in Additional file 1: Table S3.

These studies were assessed and scored according to the ROBINS-I [13], most of the review studies had a serious risk of bias, and only three studies showed a moderate risk of bias [20, 21, 23], specific details on scoring are included in Additional file 1: Fig. S6.

**Fig. 2:**
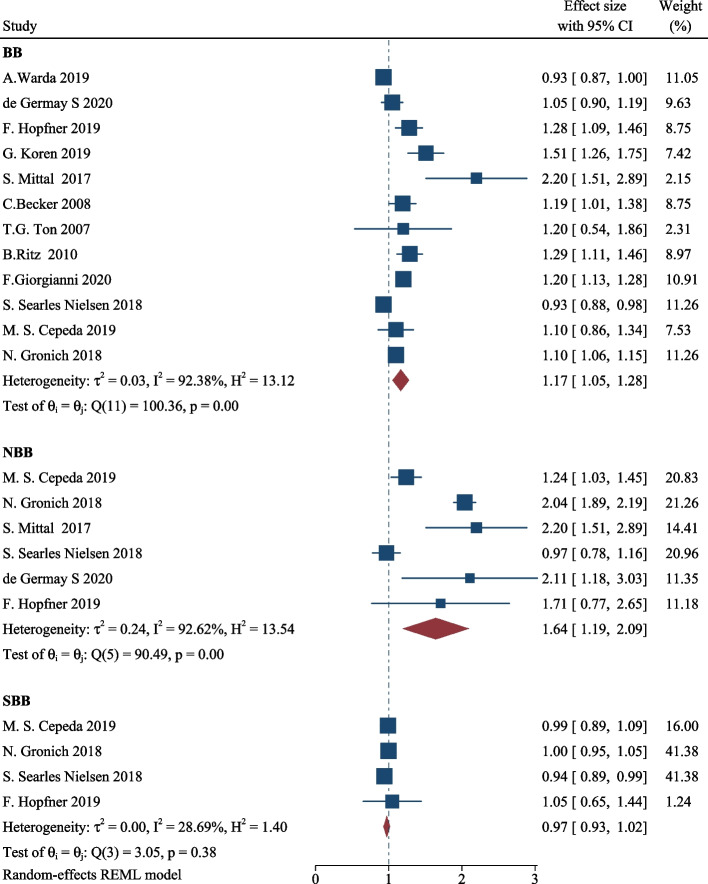
Meta-analyses of the association between the use of BBs, NBBs, SBBs, and PD. The square represents the results of a single study. The small diamond at the bottom of the forest plot represents the pooled RR, and its width represents the 95%CI. BB, beta-adrenoceptor blocker; NBB, nonselective beta-adrenoceptor blockers; SBB, selective beta-1 receptor antagonist; PD, Parkinson’s disease; CI, confidence interval; REML, Restricted Maximum Likelihood

Due to high statistical heterogeneity, a random-effects model was adopted. Figure 2 displays the forest plot illustrating the association between the use of BBs, NBBs, and SBBs with PD. BBs were associated with a risk of incident PD (RR, 1.17; 95% CI, 1.05–1.28). PD risk was further elevated in NBBs when stratified by subtypes of BBs (RR, 1.64; 95% CI, 1.19–2.09). The use of SBBs was not significantly associated with PD (RR, 0.97; 95% CI, 0.93–1.02). Due to the observational nature of these studies, the evidence on the associations between BBs and the development of PD was of very low quality according to the GRADE rating system (Additional file 1: Table S4). In this sensitivity analysis, we did not observe a great change in RR, which proved that heterogeneity does not come from a single article and our analysis results are robust. (Additional file 1: Fig. S7).

### Cohort study in UK biobank

A total of 105,763 participants (mean [SD] age, 60.7 [6.48] years; 53.7% female) were assessed in this cohort study. The basic characteristics of the patients are displayed in Table 1.

**Table 1:** Characteristics of participants at baseline. BB, beta-adrenoceptor blocker; NBB, nonselective beta-adrenoceptor blockers; SD, Standard deviation; BMI, Body mass index

Characteristic	Treated with BBs (*n* = 33,180)	Treated without BBs (*n* = 72,583)	Treated with NBBs (*n* = 4929)	Treated without NBBs (*n* = 100,834)
Age at recruitment (years), mean (SD)	61.0(6.5)	60.6(6.5)	58.5(7.6)	60.8(6.4)
Sex, %				
Female	47.9	42.9	59.9	45.7
Male	52.1	57.1	40.1	54.3
BMI (kg/m^2^), mean (SD)	29.5(5.2)	29.6(5.3)	28.4(5.3)	29.6(5.3)
Race				
White, %	94.9	92.8	95.3	93.4
Non-white, %	5.1	7.2	4.7	6.6
Smoking status, %				
Never or Previous	89.9	91.2	88.4	90.9
Current	7.7	6.6	9.4	6.8
Unknown	2.4	2.3	2.2	2.3
Alcohol consumption frequency, %				
None	11.2	10.0	13.9	10.2
Special occasions only	13.7	13.7	15.4	13.6
1–3 Times a mouth	10.7	10.7	12.1	10.5
1–2 Times a week	24.1	24.1	22.1	24.0
3–4 Times a week	20.3	20.3	17.9	20.8
Daily or almost daily	19.8	19.8	18.7	21.0
Socioeconomic status (Townsend deprivation score), mean (SD)	− 1.0(3.2)	− 1.1(3.2)	− 1.0(3.2)	− 1.1(3.2)
Incomes score, mean (SD)	0.13(0.1)	0.12(0.1)	0.12(0.1)	0.13(0.1)
Health score, mean (SD)	0.05(0.9)	− 0.02(0.8)	0.02(0.9)	0.01(0.9)
Education score, mean (SD)	18.5(17.7)	17.1(17.0)	17.5(17.3)	17.5(17.2)
Anxiety, %	59.5	57.7	68.4	57.8
Diabetes,%	8.3	7.0	4.9	7.6
Hyperlipidemia,%	19.7	8.2	8.5	12.0
Cerebrovascular diseases,%	2.5	2.1	1.9	2.2
Hypertension,%	54.0	55.7	35.6	56.1

**Fig. 3:**
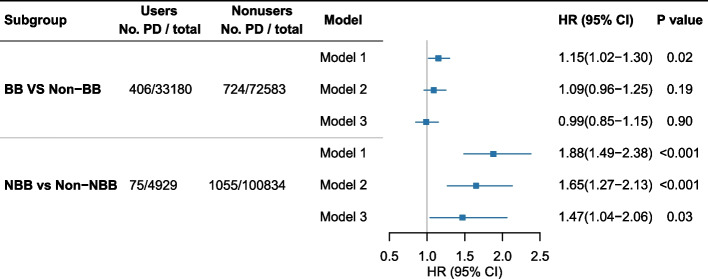
Association between the use of BBs, NBBs, SBBs, and PD in the UK Biobank. Model 1 was adjusted by age at recruitment, sex, and ethnicity; Model 2 was adjusted by smoking status, alcohol use, BMI, anxiety, socioeconomic status (Townsend deprivation score), income level, and educational level; Model 3 further adjusted for hypertension, the duration of hypertension, diabetes, hyperlipidemia, cerebrovascular diseases and the PD PRS. BB, β-blocker; NBB, nonselective beta-adrenoceptor blockers; PD, Parkinson’s disease; HR, hazard ratio; CI, confidence interval

During a total of 1,297,332 person-years and a median of 12.8 (IQR, 11.9–13.6) years of follow-up, we documented 1130 cases of PD in participants. No significant association between BBs and an increased risk of PD was found, while an association between NBB use and an increased risk of PD was observed. In Model 1, participants who regularly used NBBs had an 88% higher risk of PD than nonusers (HR, 1.88; 95% CI, 1.49–2.38). This association persisted in Model 2 after adjustment for sociodemographics, lifestyle factors, and anxiety (HR, 1.65; 95% CI, 1.27–2.13). Even in the fully adjusted model (Model 3), the results showed no major change (HR, 1.47; 95% CI, 1.04–2.06) (Fig. 3).

The sensitivity analyses supported these main findings. Results showed no significant change in the associations between NBB use and incident PD when we excluded participants who developed PD outcomes within the first year of follow-up (HR, 1.42; 95% CI, 1.00–2.00). Additionally, the results were unchanged when we excluded patients with tremors before the date of PD diagnosis (HR, 1.51; 95% CI 1.08–2.12). Furthermore, no major changes were observed in the association between NBBs and PD after adjusting no. of long-term conditions (HR, 1.41; 95% CI, 1.01–1.98), and using a competing risk regression (HR, 1.53; 95% CI, 1.02–2.29) (Additional file 1: Fig. S8).

### MR analysis

**Fig. 4:**
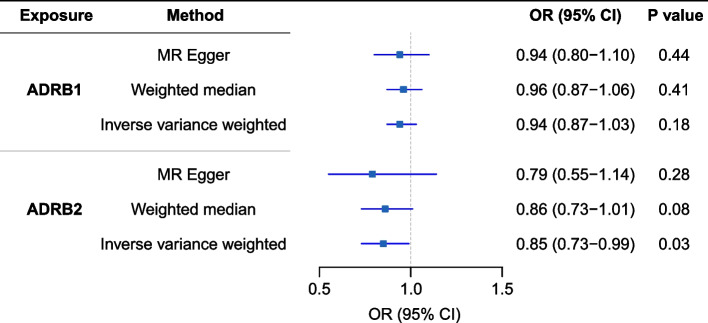
MR association between ADRB1 or ADRB2 and PD risk. ADRB1, β1 adrenergic receptor; ADRB2, β2 adrenergic receptor; CI, confidence interval; MR, Mendelian randomization; OR, odds ratio; PD, Parkinson’s disease

We found a decreased PD risk with the higher expression of the β2 adrenergic receptor (ADRB2) gene in blood with the results of IVW as the primary causal effect estimates (OR, 0.85; 95% CI, 0.73–0.99), while no association between β1 adrenergic receptor (ADRB1) gene expression and PD risk was found (OR, 0.94; 95% CI, 0.87–1.03)(Fig. 4). These results further validate our findings regarding the association between NBB and the risk of Parkinson’s disease.

## Discussion

This study reports the most comprehensive assessment of the association between NBB use and PD development by analysis of the pharmacovigilance dataset, meta-analysis, the UK Biobank dataset, and MR analysis. The FAERS database, which may be subject to reporting bias, precisely directed our research focus toward NBBs when investigating the association between commonly used antihypertensive drugs and the onset of PD. While the meta-analysis and UK Biobank Cohort analysis provided a higher level of evidence for this association. Our meta-analysis of 12 studies demonstrated that NBB use was associated with a 64% increased risk of PD compared with nonusers. The result of the UK Biobank Cohort analysis was similar to the result of the meta-analysis. MR analysis identifies the long-term modulation of ADRB2 on PD risk, which further explains the possible targets of NBB triggering PD risk. Using multiple data sources, this study has consistently shown an association between the use of NBBs and an increased risk of PD.

The association between BB exposure and the development of PD has been found in many epidemiological studies. A study including 145,098 patients who received BBs, and 1,187,151 nonusers, showed that BB users had 1.51 times the risk of incident PD compared with nonusers [22]. However, the results were inconsistent, in a case–control analysis conducted using the General Practice Research Database in the UK, it was observed that the utilization of BBs did not significantly modify the PD risk (OR, 1.16; 95% CI 0.95–1.41) [23]. The results of our study indicate that an elevated risk of PD was not observed across all BBs, but rather limited to NBBs. Consistent with our research, Gronich et al. conducted a nested case–control study involving a cohort of 1,762,164 individuals. Their investigation similarly revealed variations in the risk of PD across different subtypes of BBs, with the utilization of NBBs being linked to an elevated risk of PD (RR, 2.04; 95% CI, 1.90–2.20) [29]. In addition, a nested case–control study involving 2225 newly diagnosed PD patients revealed a lack of significant association between PD and BBs (OR, 1.05; 95%CI, 0.91–1.20), except for propranolol, an NBB (OR, 2.11; 95%CI, 1.38–3.23) [20].

Several studies suggest that the increase in the risk of PD associated with NBB use can be explained by reverse causation. For instance, Hopfner et al. proposed that the increased risk of PD could be attributed to protopathic bias, as prodromal PD commonly presents with a non-specific action tremor that is often treated with NBBs, particularly propranolol [21]. In our sensitivity analyses, the findings remained consistent even after excluding patients who exhibited tremors before the date of Parkinson’s disease diagnosis. Similarly, a secondary analysis by Gronich et al. restricted the patient population to those without essential tremors at baseline and found that the effect remained significant (RR for propranolol 1.90; 95% CI 1.72–2.09) [29]. To augment the reliability of our findings, we conducted an MR analysis. Noteworthy aspects of this analysis encompass the utilization of genetic variants within genes responsible for encoding drug targets as proxies for the potential impact of BBs. This approach effectively mitigates confounding factors and eliminates the possibility of reverse causation bias. The MR analysis revealed a significant reduction in the risk of PD associated with elevated expression of the ADRB2 gene in blood samples. Conversely, no correlation was observed between the expression of the ADRB1 gene and the risk of developing PD.

These findings are supported by mechanistic research. In human SK-N-MC neuroblastoma cells, it has been demonstrated that ADRB2 agonists decrease the abundance of SNCA mRNA and the production of alpha-synuclein protein. Conversely, ADRB2 antagonists have been shown to increase the expression of SNCA, leading to increased oxidative stress in mitochondria, dopaminergic neurodegeneration, and an elevated risk for PD [9]. It is important to note that SBBs specifically target ADRB1 receptors without affecting ADRB2 receptors. While NBBs block both ADRB1 and ADRB2 receptors. This provides a more comprehensive justification for our findings in observational studies, which demonstrated a higher risk of developing PD associated with the use of NBBs rather than SBBs.

Hypertension has been identified as a risk factor for PD [30]. However, the current evidence does not support the idea that existing antihypertensive medications can effectively reduce the risk of PD. Even in our study, NBBs were associated with increased risk for PD. In light of this, it becomes even more meaningful to focus on preventive measures targeting hypertension through lifestyle modifications and other non-pharmacological approaches. On the other hand, individuals with pre-existing hypertension, especially those with additional risk factors for PD, should consider alternative antihypertensive treatment options whenever possible, due to the potential risk of PD associated with NBBs.

### Strengths and limitations

There are several strengths in our study. Analyzing real-world pharmacovigilance data provides an additional information source. Meta-analysis can make significant contributions to issues by risk for various antihypertensive drugs combining the results from current epidemiological studies. Due to the large number of participants and cases in the UK Biobank data, we were able to compare the risk of developing PD with an active control and evaluate the association between various antihypertensive drugs and PD development. In addition, our data were collected before diagnosis, avoiding potential recall and selection biases as well as misclassification during follow-up. Finally, robust sensitivity analyses also increased our confidence in the results. More importantly, we observed consistent associations between NBBs and the risk of PD across 3 independent approaches from the meta-analysis, pharmacovigilance database, and UK biobank. With the uniform results, Mendelian randomization analysis which employed genetic variation as an instrumental variable to discover and quantify causation was also used, thereby overcoming the impact of possible confounding and reverse causality.

This study had some limitations that need to be considered in the interpretation of the findings. First, it was an observational study based on multiple sources, therefore, reverse causality might exist. However, the study rigorously adjusted for confounding factors and validated the association through Mendelian randomization analysis, thereby addressing this issue to the best extent possible. Second, in the FAERS data, the reported PD may have also been owing to other reasons aside from the administration of antihypertensive agents despite our limiting the analysis to participants with hypertension. Third, there was substantial statistical heterogeneity among the included studies in our meta-analysis which must be noted even though we used a random-effects model to pool the effect estimates and reported subgroup analysis to explore heterogeneity. Fourth, there was a chance of misclassification of antihypertensive agents used in the UK Biobank data during follow-up because antihypertensive agent use was only evaluated once at baseline. Fifth, as the UK Biobank did not collect information on the use of antihypertensive agents (i.e., dosage, frequency, and duration), we could not explore the possible dose–response relationship.

## Conclusions

Overall, our comprehensive study indicated that regular NBB use is associated with an increased risk of PD. Some should choose alternative antihypertensive treatment options when possible given the potential risk.

### Supplementary Information

**Additional file 1:** **Text S1.** Polygenic risk score for PD. **Table S1.** Literature search strategy. **Table S2.** List of the disease conditions included in multimorbidity count. **Table S3.** Summary of Included Studies. **Table S4.** Grading of evidence included in meta-analysis. **Fig. S1.** Study design. **Fig. S2.** Flow chart of included participants. **Fig. S3.** R^2^ of polygenic risk score (PRS) by different P value thresholds. **Fig. S4.** Association of the polygenic risk score (PRS) for PD with incident PD using Cox proportional hazards regression. **Fig. S5.** Flow chart of included literature. **Fig. S6.** Summary of Risk of Bias Using ROBINS-I. **Fig. S7.** Results of leave-one-out analysis. **Fig. S8.** Results of sensitivity analyses

## Data Availability

The data were available from the UK Biobank Resource (www.ukbiobank.ac.uk/). This research has been conducted using the UK Biobank Resource under Application Number 75283. The pharmacovigilance data used in this study are available from the FAERS website (https://fis.fda.gov/extensions/FPD-QDE-FAERS/FPD-QDE-FAERS.html) and were cleaned and normalized using the Open Vigil 2 tool (http://openvigil.sourceforge.net/).

## Abbreviations

ACEI: Angiotensin-converting enzyme inhibitor
ARB: Angiotensin receptor blocker
BB: Beta-adrenoceptor blocker
CCB: Calcium channel blocker
FAERS: Food and Drug Administration Adverse Event Reporting System
MedDRA: Medical Dictionary for Regulatory Activities
MR: Mendelian randomization
NBB: Nonselective beta-adrenoceptor blocker
PD: Parkinson’s disease
SBB: Selective β1-adrenoceptor blocker
