# Supplementary material for: Nonselective beta-adrenoceptor blocker use and risk of Parkinson’s disease: from multiple real-world evidence
Source: BMC Med. 2023 Nov 14;21:437. doi: 10.1186/s12916-023-03122-z (PMC10647086; doi:10.1186/s12916-023-03122-z)

**Additional file 1**

**Table of Contents**

**Text S1. Polygenic risk score for PD**

**Table S1. Literature search strategy.**

**Table S2 List of the disease conditions included in multimorbidity count.**

**Table S3. Summary of Included Studies.**

**Table S4. Grading of evidence included in meta-analysis.**

**Fig. S1. Study design.**

**Fig. S2 Flow chart of included participants.**

**Fig. S3. R^2^ of polygenic risk score (PRS) by different P value thresholds**

**Fig. S4.** **Association of the polygenic risk score (PRS) for PD with incident PD using Cox proportional hazards regression.**

**Fig. S5. Flow chart of included literature.**

**Fig. S6. Summary of Risk of Bias Using ROBINS-I.**

**Fig. S7. Results of leave-one-out analysis.**

**Fig. S8.** **Results of sensitivity analyses.**

**Text S1. Polygenic risk score for PD**

We assessed the genetic susceptibility to PD by utilizing the polygenic risk score (PRS), which measures the combined influence of common genetic variants on the disease's onset. The PRS was calculated using GWAS summary data obtained from the FinnGen consortium (available at https://storage.googleapis.com/finngen-public-data-r8/summary_stats/finngen_R8_G6_ PARKINS ON.gz). The FinnGen GWAS for PD was conducted in 3767 cases and 338732 controls, with all participants of European ancestry and no sample overlap with UK Biobank. Genotype data from UKB were used as the target data. We generated PRS for PD risk using PRSice-2.1 The PRS was computed at P value thresholds from 1 to 0.00075 of the base data, and linkage disequilibrium clumping was performed in the target data using the default parameter setting. The default model of the PRS algorithm was selected, which involves counting the number of observed effect alleles for each variant, multiplying it by the corresponding effect size, and dividing it by the total number of alleles included in the PRS for each individual. The results of all variants are then summed for each individual. The transferability and predictability of these PRSs were assessed by evaluating their P values and R^2^ values, respectively, adjusting for age, sex, and population stratification (top ten principal components). The best-fitted model for PRS was identified.( Additional file 1. Fig S3)

The PRS was standardized to z-scores (PRS-z) based on the mean and standard deviation observed in the samples. Elevated scores signify an augmented genetic susceptibility to Parkinson's disease (PD). Additionally, we classified the PRS-Z into quartiles, with the first quartile serving as the reference group representing the lowest genetic risk. To validate the PRS-z, we evaluated its predictive capacity for PD, considering it both as a continuous and categorical variable. Notably, a strong association between the PRS-Z and PD was observed. (Additional file 1: Fig S4).

**Table S1. Literature search strategy.**

| Database | Search terms |
| --- | --- |
| **PubMed** | #1 ("Adrenergic beta-Antagonists"[Mesh]) OR (Adrenergic beta Antagonists OR beta-Antagonists, Adrenergic OR Adrenergic beta-Receptor Blockader OR Adrenergic beta Receptor Blockader OR Blockader, Adrenergic beta-Receptor OR beta-Receptor Blockader, Adrenergic OR beta-Adrenergic Antagonist OR Antagonist, beta-Adrenergic OR beta Adrenergic Antagonist OR beta-Adrenergic Blocker OR Blocker, beta-Adrenergic OR beta Adrenergic Blocker OR beta-Adrenergic Antagonists OR Antagonists, beta-Adrenergic OR beta Adrenergic Antagonists OR beta-Adrenoceptor Antagonists OR Antagonists, beta-Adrenoceptor OR beta Adrenoceptor Antagonists OR Adrenergic beta-Receptor Blockaders OR Adrenergic beta Receptor Blockaders OR Blockaders, Adrenergic beta-Receptor OR beta-Receptor Blockaders, Adrenergic OR beta-Adrenergic Receptor Blockaders OR Blockaders, beta-Adrenergic Receptor OR Receptor Blockaders, beta-Adrenergic OR beta Adrenergic Receptor Blockaders OR beta-Adrenergic Blocking Agents OR Agents, beta-Adrenergic Blocking OR Blocking Agents, beta-Adrenergic OR beta Adrenergic Blocking Agents OR beta-Adrenergic Blockers OR Blockers, beta-Adrenergic OR beta Adrenergic Blockers OR beta-Blockers, Adrenergic OR Adrenergic beta-Blockers OR beta Blockers, Adrenergic OR beta-Adrenergic Blocking Agent OR Agent, beta-Adrenergic Blocking OR Blocking Agent, beta-Adrenergic OR beta Adrenergic Blocking Agent OR beta-Adrenergic Receptor Blockader OR Blockader, beta-Adrenergic Receptor OR Receptor Blockader, beta-Adrenergic OR beta Adrenergic Receptor Blockader OR beta-Adrenoceptor Antagonist OR Antagonist, beta-Adrenoceptor OR beta Adrenoceptor Antagonist OR Adrenergic beta-Antagonist OR Adrenergic beta Antagonist OR beta-Antagonist, Adrenergic)  #2 ("Parkinson Disease"[Mesh]) OR (Idiopathic Parkinson's Disease OR Lewy Body Parkinson's Disease OR Parkinson's Disease, Idiopathic OR Parkinson's Disease, Lewy Body OR Parkinson Disease, Idiopathic OR Parkinson's Disease OR Idiopathic Parkinson Disease OR Lewy Body Parkinson Disease OR Primary Parkinsonism OR Parkinsonism, Primary OR Paralysis Agitans)  #3 #1 AND #2  Period of inclusion: to December 31, 2022 |
| EMBASE | #1 'beta adrenergic receptor blocking agent'/exp  #2 'beta adrenergic receptor blocking agent':ab,ti OR 'adrenergic beta antagonists':ab,ti OR  'adrenergic beta-antagonists':ab,ti OR 'antiadrenergics, beta blocking':ab,ti OR 'beta adrenergic antagonist':ab,ti OR 'beta adrenergic blocker':ab,ti OR 'beta adrenergic blockers':ab,ti OR 'beta adrenergic blocking agent':ab,ti OR 'beta adrenergic blocking drug':ab,ti OR 'beta adrenergic receptor antagonist':ab,ti OR 'beta adrenergic receptor blocker':ab,ti OR 'beta adrenoceptor antagonist':ab,ti OR 'beta adrenoceptor blocker':ab,ti OR 'beta adrenoceptor blocking agent':ab,ti OR 'beta adrenoceptor blocking drug':ab,ti OR 'beta adrenolytic':ab,ti OR 'beta adrenolytic agent':ab,ti OR 'beta antagonist':ab,ti OR 'beta antiadrenergic agent':ab,ti OR 'beta blocker':ab,ti OR 'beta blocking adrenergic agent':ab,ti OR 'beta blocking agent':ab,ti OR 'beta blocking drug':ab,ti OR 'beta receptor adrenergic blocking agent':ab,ti OR 'beta receptor blocker':ab,ti OR 'beta receptor blocking agent':ab,ti OR 'beta sympathicolytic agent':ab,ti OR 'beta sympathicolytics':ab,ti OR 'beta sympatholytic agent':ab,ti OR 'betasympatholytic agent':ab,ti  #3 #1 OR #2  #4 ' Parkinson's disease'/exp  #5 'idiopathic parkinsons disease':ab,ti OR 'parkinson disease':ab,ti OR 'parkinsons disease, idiopathic':ab,ti OR 'Parkinsons Disease, Lewy Body':ab,ti OR 'Parkinson Disease, Idiopathic':ab,ti OR 'Parkinsons Disease':ab,ti OR 'Idiopathic Parkinson Disease':ab,ti OR 'Lewy Body Parkinson Disease':ab,ti OR 'Primary Parkinsonism':ab,ti OR 'Parkinsonism, Primary':ab,ti OR 'Paralysis Agitans':ab,ti #6 #4 OR #5  #7 #3 AND #6  Period of inclusion: to December 31, 2022 |
| **Web of Science** | #1 ((((TS=(beta-Antagonist)) OR TS=(beta-Antagonist)) OR TS=(beta blocker)) OR TS=(beta-blocker)) OR TS=(beta Adrenergic Antagonist) #2 ((TS=(Parkinson disease)) OR TS=(Parkinson's disease)) OR TS=(Parkinson*)  #3 #1 AND #2  Period of inclusion: to December 31, 2022 |

**Table S2** List of the disease conditions included in multimorbidity count.

|  | Condition | ICD-10 |
| --- | --- | --- |
| 1 | Hypertension diseases | I10, I11, I12, I13, I14, I15 |
| 2 | Coronary heart disease | I20, I21, I22, I23, I24, I25 |
| 3 | Diabetes | E10, E11, E12, E13, E14 |
| 4 | Cerebrovascular diseases | I60, I61, I62, I63, I64, I65, I66, I67, I68, I69 |
| 5 | Atrial fibrillation and flutter | I48 |
| 6 | Heart failure | I50 |
| 7 | Peripheral vascular disease | I70, I71, I72, I73, I74, I75, I76, I77, I78, I79 |
| 8 | COPD | J44 |
| 9 | Asthma | J45 |
| 10 | Bronchiectasis | J47 |
| 11 | Cancer | C00-C97 |
| 12 | Dyspepsia | K30 |
| 13 | Diverticular disease | K57 |
| 14 | Irritable bowel syndrome | K58 |
| 15 | Diseases of liver | K70, K71, K72, K73, K74, K75, K76, K77 |
| 16 | Inflammatory bowel disease | K50, K51 |
| 17 | Constipation | K59.0 |
| 18 | Viral hepatitis | B15, B16, B17, B18, B19 |
| 19 | Depression | F32 |
| 20 | Anxiety | F40, F41 |
| 21 | Schizophrenia/Bipolar  affective disorder | F20, F30, F31 |
| 22 | Connective tissue diseases | M30, M31, M32, M33, M34, M35, M36 |
| 23 | Painful conditions | M54, M25.5, R51, M53, M50, M72.2, G56.0, M79.7, M05, B02, M47, G50.0, M10 |
| 24 | Osteoporosis | M80, M81, M82 |
| 25 | Thyroid disorders | E01, E02, E03, E04, E05, E06, E07 |
| 26 | Alcohol problems | F10, K70 |
| 27 | Chronic kidney disease | N18, Q61 |
| 28 | Prostate disorders | N40, N41 |
| 29 | Glaucoma | H40, H41, H42 |
| 30 | Epilepsy | G40 |
| 31 | Dementia | G30, F00, F01, F02, F03 |
| 32 | Psoriasis or eczema | L20, L40 |
| 33 | Migraine | G43 |
| 34 | Chronic sinusitis | J32 |
| 35 | Anorexia or bulimia | F50.0, F50.2 |
| 36 | Multiple sclerosis | G35 |
| 37 | Chronic fatigue syndrome | G93.3 |
| 38 | Endometriosis | N80 |
| 39 | Meniere disease | H81.0 |
| 40 | Pernicious anaemia | D51.0 |
| 41 | Polycystic ovaries | E28.2 |

ICD, International Classification of Disease

**Table S3.** Summary of Included Studies.

| **Source/Year** | **Location** | **Design** | **Database/source** | **Cohort size** | **Study duration** |
| --- | --- | --- | --- | --- | --- |
| B.Ritz 2010 | Denmark | case–control study | prescriptions from pharmacies in Denmark | With PD: 1931; Without PD: 9651 | 2001-2006 |
| F.Giorgianni 2020 | United Kingdom | case–control study | UK Clinical Practice Research Datalink | With PD: 8604; Without PD: 86040 | 1995-2016 |
| C.Becker 2008 | United Kingdom | case–control study | Exposure ascertained based on current or past prescription record. | With PD: 3637; Without PD: 3637 | 1994-2005 |
| T.G. Ton 2007 | USA | case–control study | GHC pharmacy database | With PD: 206; Without PD: 383 | 1992–2002 |
| S.D.Germay 2020 | France | case–control study | National Health Data System (SNDS) General Sample of Beneficiaries (EGB) | With PD: 2225; Without PD: 2225 | 2008–2017 |
| A.Warda 2019 | Germany | case–control study | Disease Analyzer database of IQVIA | With PD: 9127; Without PD: 9127 | 2013–2017 |
| M.S.Cepeda 2019 | USA | self-controlled cohort study | US claims databases | BB users: 5789762 | 2000-2018 |
| G.Koren 2019 | Israel | cohort study | Electronic medical records (EMRs) of Maccabi Health Services | BB users: 145098; BB non-users: 1187151 | 1998–2004 |
| F.Hopfner 2019 | Denmark | case–control study | Danish National Registry of Patients, the Danish National Prescription Registry, and the Danish Person Registry | With PD: 2790; Without PD: 11160 | 2000–2012 |
| N.Gronich 2018 | Israel | case–control study | EMR database of Clalit Health Services (CHS) | With PD: 11314; Without PD: 113140 | 2004–2017 |
| S.S.Nielsen 2018 | USA | case–control study | United States Medicare Data | With PD: 48295; Without PD: 52324 | 2004–2009 |
| S.Mittal 2017 | Norway | case–control study | Norwegian National Registry (NNR), Norwegian Prescription Database (NorPD)  and Norwegian National Education Database (NNED) | BB users: 14794; BB non-users: 4671188 | 2004–2015 |

**Table S4. Grading of evidence included in meta-analysis.**

| **Certainty assessment** | | | | | | | **№ of patients** | | **Effect** | | **Certainty** |
| --- | --- | --- | --- | --- | --- | --- | --- | --- | --- | --- | --- |
| **№ of studies** | **Study design** | **Risk of bias** | **Inconsistency** | **Indirectness** | **Imprecision** | **Other considerations** | **Intervention** | **Comparison** | **Relative (95% CI)** | **Absolute (95% CI)** |  |
| Risk of developing PD in BB users compared to non-users | | | | | | | | | | | |
| 12 | observational studies | not serious | serious ^a^ | not serious | serious | none | 88129 cases 287687controls; 5949654 exposed 5858339 unexposed | | **RR 1.17** (1.05 to 1.28) | - | ⨁◯◯◯ VERY LOW |
|  |  |  |  |  |  |  | - | 0.0% |  | **0 fewer per 100,000** (from 0 fewer to 0 fewer) |  |
| Risk of developing PD in NBB users compared to non-users | | | | | | | | | | | |
| 6 | observational studies | not serious | serious ^a^ | not serious | serious ^b^ | none | 64624 cases 176624 controls; 1560259 exposed 4671188 unexposed | | **RR 1.64** (1.19 to 2.09) | - | ⨁◯◯◯ VERY LOW |
|  |  |  |  |  |  |  | - | 0.0% |  | **0 fewer per 100,000**  (from 0 fewer to 0 fewer) |  |
| Risk of developing PD in SBB users compared to non-users | | | | | | | | | | | |
| 4 | observational studies | not serious | serious | not serious | serious ^c^ | none | 86279 cases 282699 controls 41/14794 exposed 4593/4671188 unexposed | | **RR 0.97**  (0.93 to 1.02) | **-** |  |
|  |  |  |  |  |  |  | - | 0.0% |  | **0 fewer per 100,000**  (from 0 fewer to 0 fewer) | ⨁◯◯◯ VERY LOW |

BB: beta-adrenoceptor blockers; NBB:nonselective beta-adrenoceptor blockers; SBB: selective β1-adrenoceptor blockers ; PD, Parkinson's Disease; RR, relative risk; 95%CI, 95% confidence interval.


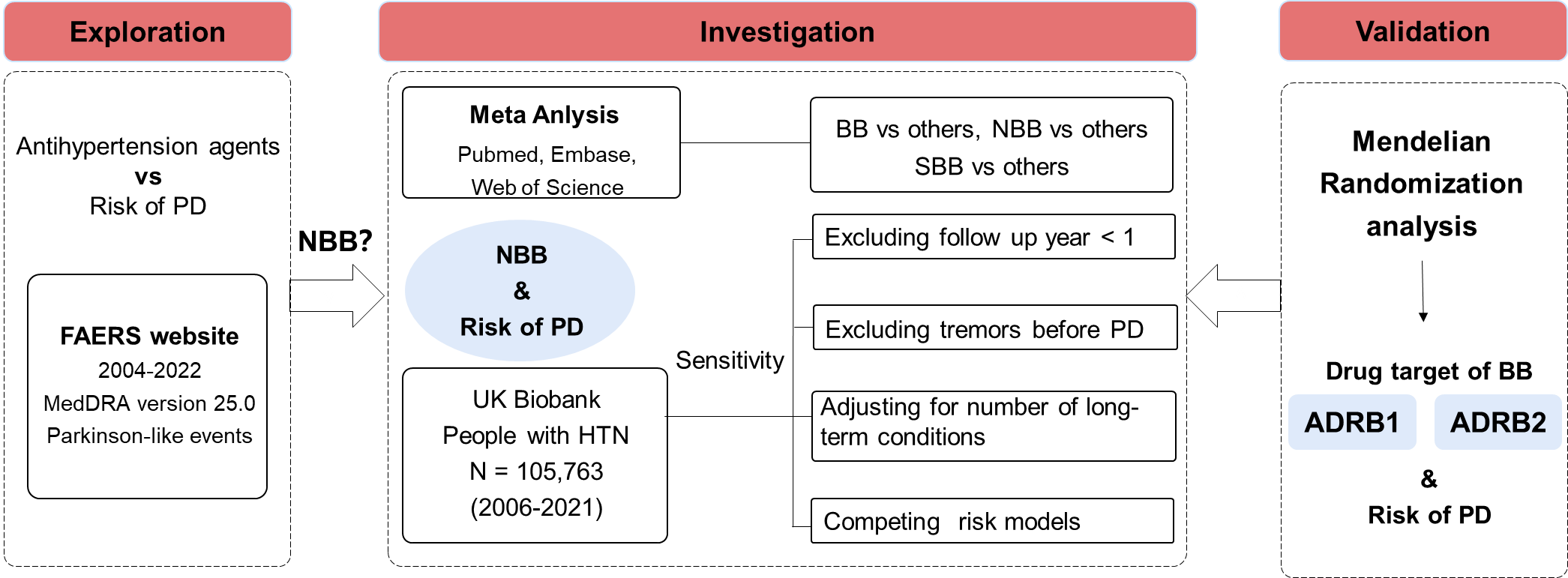
**Fig. S1. Study design.** BB: beta-adrenoceptor blockers; NBB:nonselective beta-adrenoceptor blockers; SBB: selective β1-adrenoceptor blockers ; PD, Parkinson's Disease; ADRB1:β1 adrenergic receptor; ADRB2: β2 adrenergic recepto

**Fig. S2. Flow chart of included participants.** BB: beta-adrenoceptor blockers; NBB:nonselective beta-adrenoceptor blockers; SBB: selective β1-adrenoceptor blockers; PD, Parkinson's Disease; ACEI angiotensin-converting enzyme inhibitor; ARB; angiotensin receptor blocker; CCB: calcium channel blocker


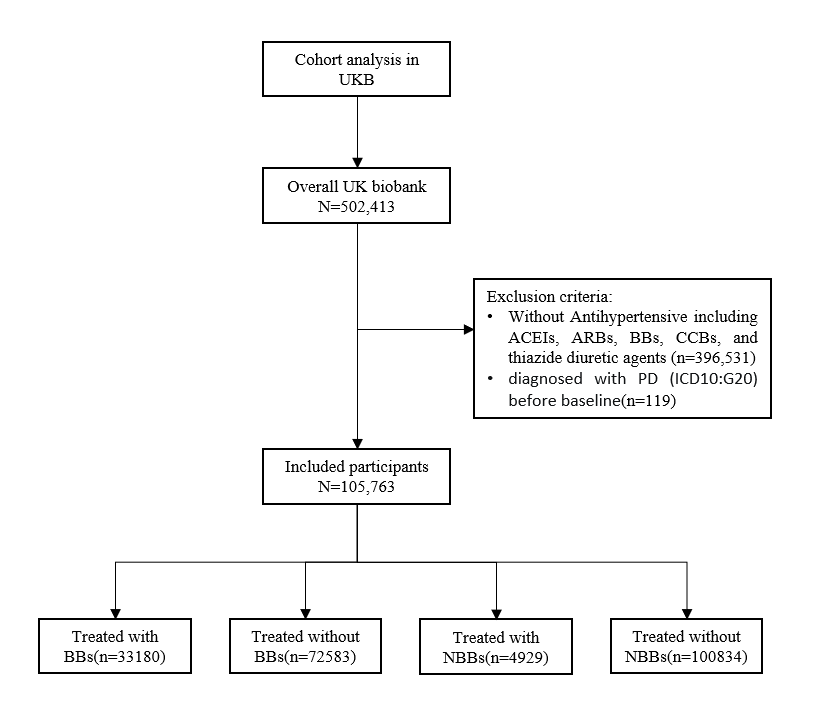


**Fig. S3. R^2^ of polygenic risk score (PRS) by different P value thresholds**

**
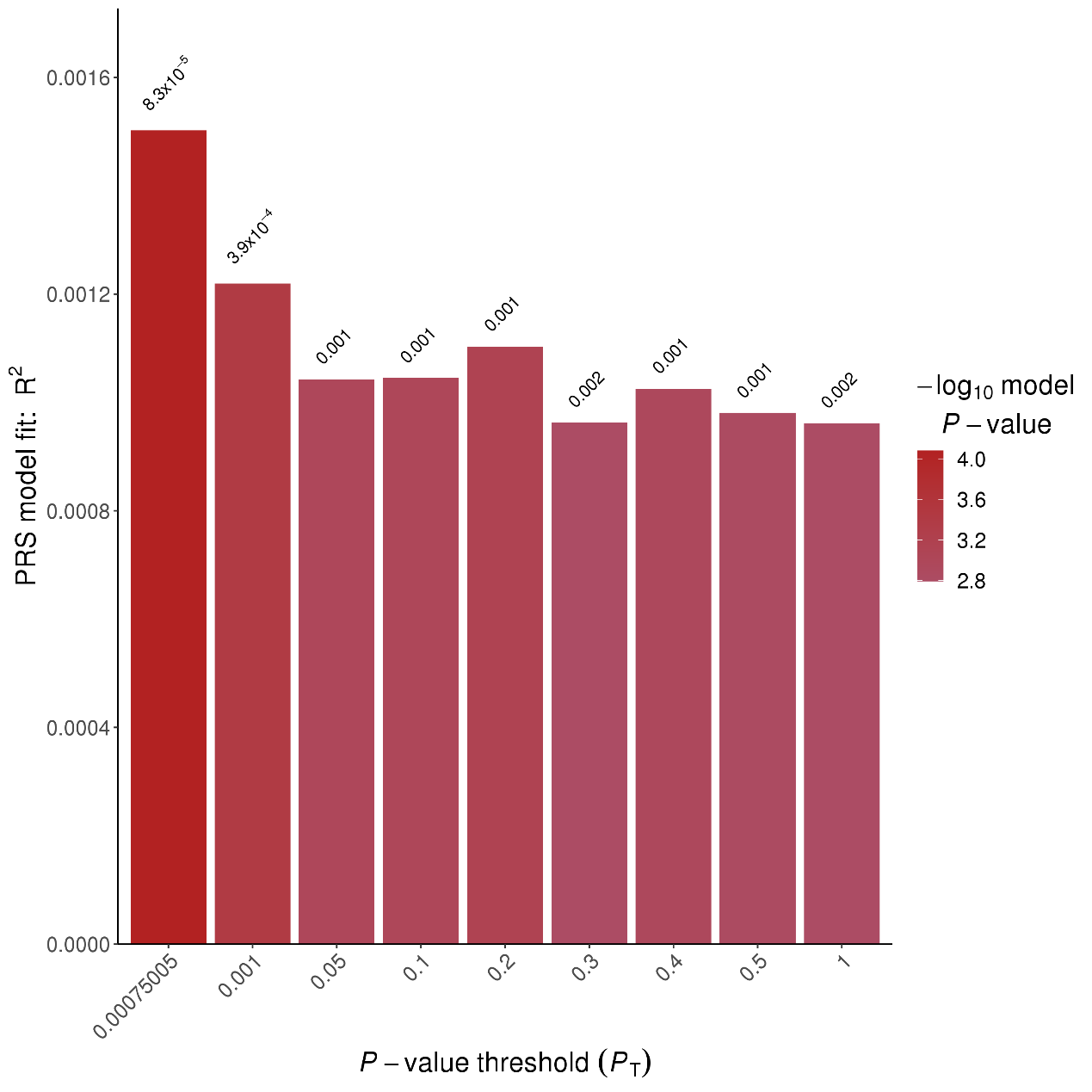
**

**Fig. S4. Association of the polygenic risk score (PRS) for PD with incident PD using Cox proportional hazards regression.** The first quartile was the reference group. HR, Hazard Ratio; CI, confidence interval


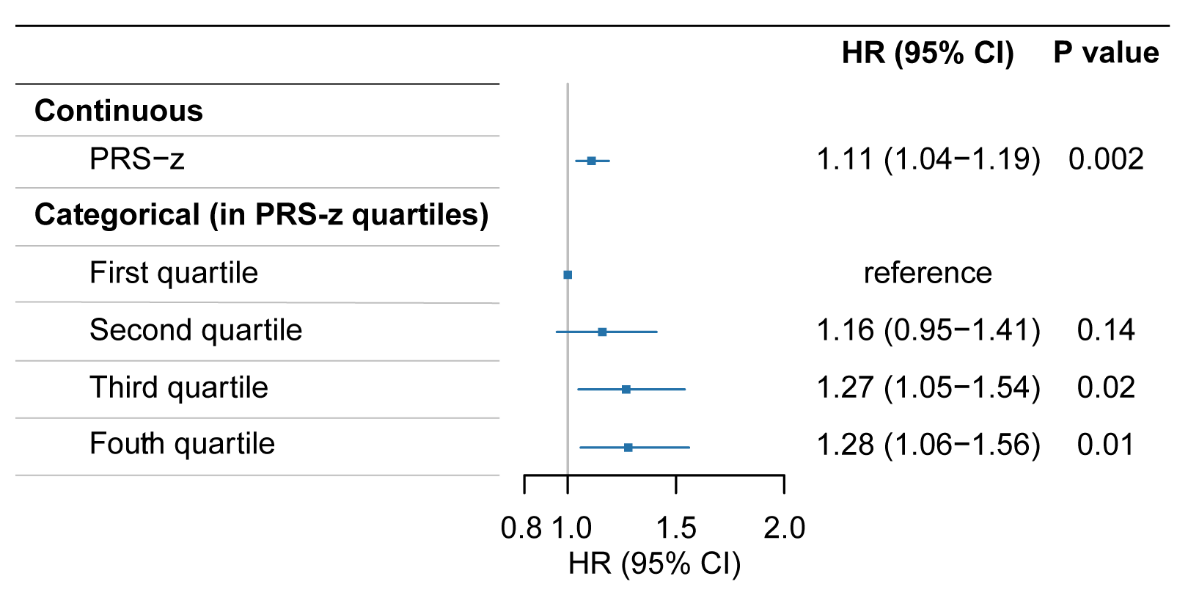


**Fig. S5.** Flow chart of included literature.

**
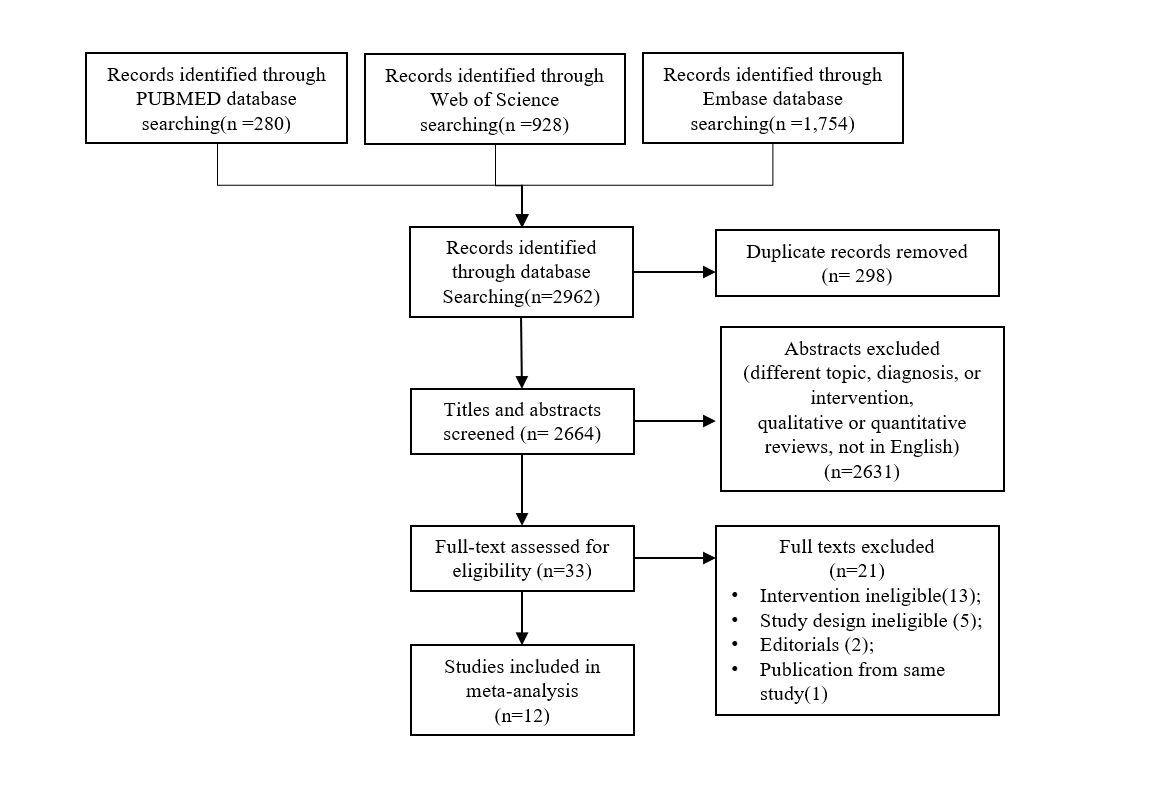
**

**Fig. S6. Summary of Risk of Bias Using ROBINS-I.** Colored bars represent the proportion of trial comparisons assessed as low (green), unclear (yellow) or high (red) risk of bias.

**
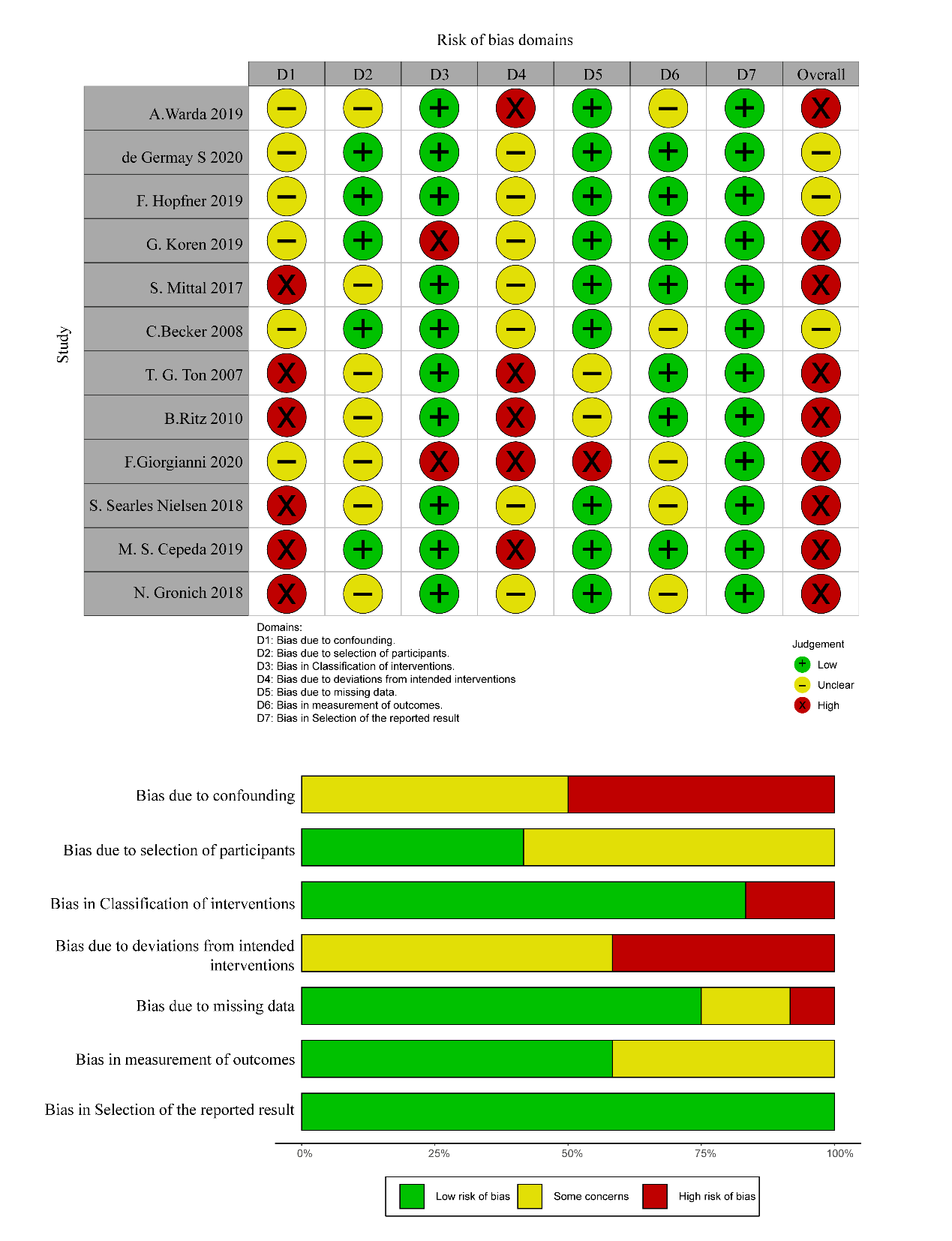
**

**Fig. S7. Results of leave-one-out analysis**. Sensitivity analysis of the systematic removal of each study comparison. (A) evaluating of an association between BB use and risk of PD (B) evaluating of an association between NBB use and risk of PD and (C) evaluating of an association between SBB use and risk of PD. RR, relative risk; 95% CI, 95% confidence interval; BB: beta-adrenoceptor blockers; NBB:nonselective beta-adrenoceptor blockers; SBB: selective β1-adrenoceptor blockers; REML, Restricted Maximum Likelihood.


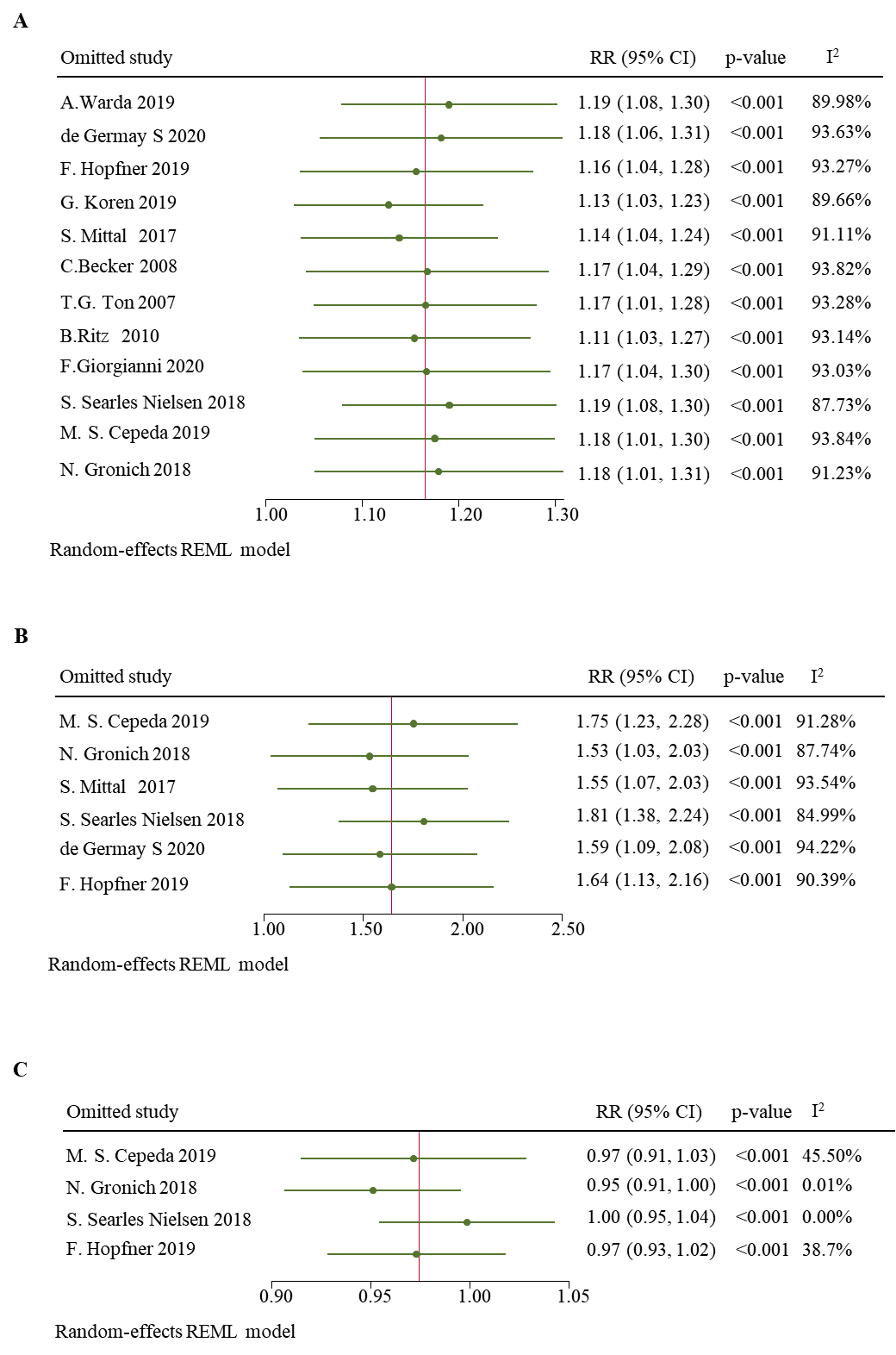


**Fig. S8.** **Results of sensitivity analyses.** PD, Parkinson's Disease; HR, hazard ratio; 95% CI, 95% confidence interval.


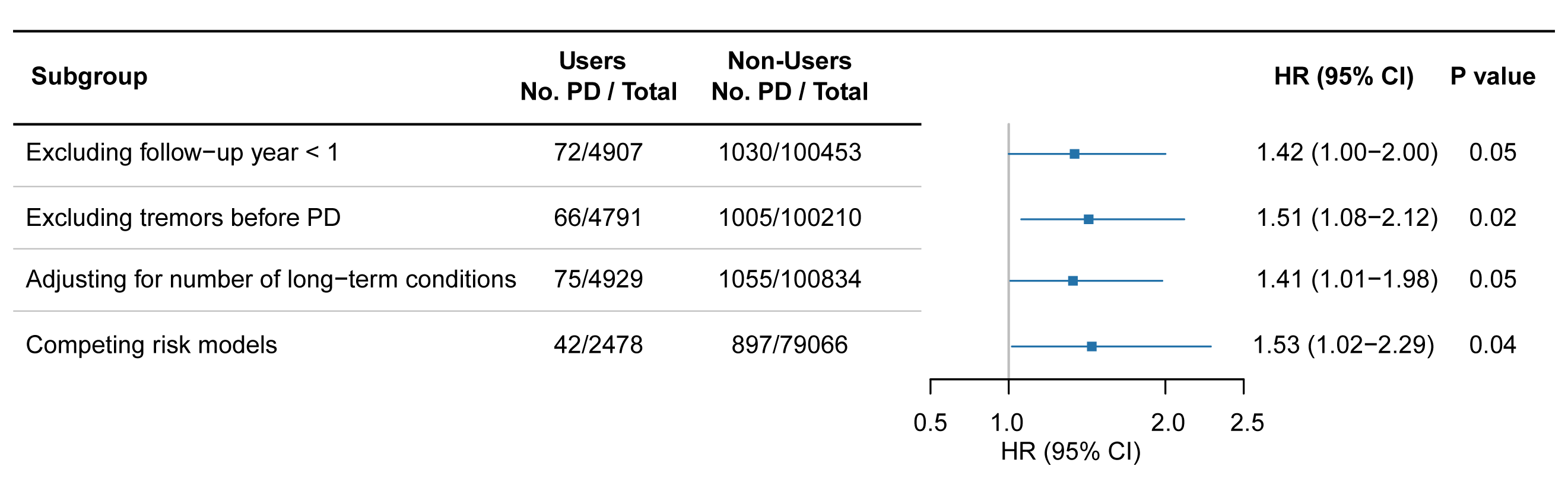

Supplement: Supplementary file 1 — Additional file 1: Text S1. Polygenic risk score for PD. Table S1. Literature search strategy. Table S2. List of the disease conditions included in multimorbidity count. Table S3. Summary of Included Studies. Table S4. Grading of evidence included in meta-analysis. Fig. S1. Study design. Fig. S2. Flow chart of included participants. Fig. S3. R2 of polygenic risk score (PRS) by different P value thresholds. Fig. S4. Association of the polygenic risk score (PRS) for PD with incident PD using Cox proportional hazards regression. Fig. S5. Flow chart of included literature. Fig. S6. Summary of Risk of Bias Using ROBINS-I. Fig. S7. Results of leave-one-out analysis. Fig. S8. Results of sensitivity analyses [file 12916_2023_3122_MOESM1_ESM.docx]
